# Assessing mental health service user and carer involvement in physical health care planning: The development and validation of a new patient-reported experience measure

**DOI:** 10.1371/journal.pone.0206507

**Published:** 2019-02-13

**Authors:** Chris J. Sidey-Gibbons, Helen Brooks, Judith Gellatly, Nicola Small, Karina Lovell, Penny Bee

**Affiliations:** 1 Patient-reported Outcomes, Value, and Experience (PROVE) Center, Brigham and Women’s Hospital, Boston, MA, United States of America; 2 Department of Surgery, Harvard Medical School, Boston, MA, United States of America; 3 Department of Psychological Sciences, Institute of Psychology, Health and Society, University of Liverpool, Liverpool, United Kingdom; 4 Mental Health Research Group, Division of Nursing, Midwifery and Social Work, Faculty of Biology, Medicine and Health, School of Health Sciences, Manchester Academic Health Science Centre, University of Manchester, Manchester, United Kingdom; 5 NIHR School of Primary Care Research, Division of Population Health, Health Services Research and Primary Care, School of Health Sciences, Faculty of Biology, Medicine and Health, Manchester Academic Health Science Centre, University of Manchester, Manchester, United Kingdom; Foundation IRCCS Neurological Institute C. Besta, ITALY

## Abstract

**Background:**

People living with serious mental health conditions experience increased morbidity due to physical health issues driven by medication side-effects and lifestyle factors. Coordinated mental and physical healthcare delivered in accordance with a care plan could help to reduce morbidity and mortality in this population. Efforts to develop new models of care are hampered by a lack of validated instruments to accurately assess the extent to which mental health services users and carers are involved in care planning for physical health.

**Objective:**

To develop a brief and accurate patient-reported experience measure (PREM) capable of assessing involvement in physical health care planning for mental health service users and their carers.

**Methods:**

We employed psychometric and statistical techniques to refine a bank of candidate questionnaire items, derived from qualitative interviews, into a valid and reliable measure involvement in physical health care planning. We assessed the psychometric performance of the item bank using modern psychometric analyses. We assessed unidimensionality, scalability, fit to the partial credit Rasch model, category threshold ordering, local dependency, differential item functioning, and test-retest reliability. Once purified of poorly performing and erroneous items, we simulated computerized adaptive testing (CAT) with 15, 10 and 5 items using the calibrated item bank.

**Results:**

Issues with category threshold ordering, local dependency and differential item functioning were evident for a number of items in the nascent item bank and were resolved by removing problematic items. The final 19 item PREM had excellent fit to the Rasch model fit (x^2^ = 192.94, df = 1515, *P* = .02, RMSEA = .03 (95% CI = .01-.04). The 19-item bank had excellent reliability (marginal r = 0.87). The correlation between questionnaire scores at baseline and 2-week follow-up was high (r = .70, *P* < .01) and 94.9% of assessment pairs were within the Bland Altman limits of agreement. Simulated CAT demonstrated that assessments could be made using as few as 10 items (mean SE = .43).

**Discussion:**

We developed a flexible patient reported outcome measure to quantify service user and carer involvement in physical health care planning. We demonstrate the potential to substantially reduce assessment length whilst maintaining reliability by utilizing CAT.

## Introduction

People diagnosed with severe mental illnesses, such as disorder schizophrenia and bipolar disorders, exhibit higher rates of physical co-morbidities and, as a result, are significantly more likely to die prematurely than the general population.[1–3]

Factors contributing to this deterioration in physical health for mental health service users are known to include side-effects from anti-psychotic medications, higher rates of smoking and substance abuse, poor nutrition, and physical inactivity.[4] Though the relationship between serious mental health issues, physical comorbidity, and reduced life expectancy is well understood, far less is known about how to organize care delivery to improve physical health and reduce the risk of associated morbidity in this population. Recent evidence suggests that, despite increased awareness of these issues, mortality risk associated with all mental health conditions is rising internationally.[2]

One approach to improve the management of known risk factors is individualized care planning; [5,6] an approach which involves service users and carers working collaboratively with professionals to co-develop a written care plan. This plan aims to accurately document the core issues that a service user would like to address as part of their mental health recovery.

A growing body of research shows that, although collaborative care planning is s aligned with the desires of both service users and carers there is a paucity of care models which have been shown to effectively increase involvement in care planning for physical health in this way.[7] More broadly, increasing the quality of mental health services was the top research priority expressed by an international working group comprising both professionals as well as users and carers.[8]

Progress in the development of interventions to improve care planning involvement between service users, carers, and providers is stymied by the lack of a meaningful outcome assessment. Quantification of abstract subjective phenomena, such as involvement with care planning, is best accomplished by directly assessing the perspective of the service user or carer; usually using a tool commonly referred to as a patient-reported outcome measure (PREM).

Patient reported outcome measures are a efficient and accurate way to quantify the views of service users and their carers. A relevant example is the EQUIP PREM, which was developed by our group to assess service user and carer involvement in mental health care planning.[9] Previous research has highlighted the importance of brief assessments for mental health service users and their carers, with a strong user preference for minimising response burden by developing shorter questionnaires.[9,10] New assessment modalities including computerized adaptive testing (CAT), are able to tailor person-centred assessments to the individual, a process which tends to result in shorter, more relevant assessments. [11]

The objective of the current paper is to create a novel PREM to assess mental health service user and carer involvement in physical health care planning. We seek to develop a PREM that is accurate, reliable, and suitable for individualized CAT assessment.

## Methods

### Item development methods

A set of 67 candidate items were developed following qualitative interviews with mental health service users (SUs), their carers, and mental health professionals from the UK. Further details of the qualitative interview process can be found in a separate manuscript.[12] Items were developed to reflect six pre-identified themes; three of which covered general mental health care planning requirements and three of which were unique to physical health care planning. The general themes included: tailoring a collaborative working relationship between the service users and their carers and the service providers, maintaining a trusting relationship with a professional, having access to a tangible document which could be edited and updated. The physical health themes were: valuing physical health equally with mental health, experiencing coordinated care between health professionals in different disciplines, and having a personalised physical heath discussion.

### Data collection

Potential participants who expressed an interest in taking part in the study were given a participant information sheet written to current UK National Research Ethics Service (NRES) guidelines. We worked with service users and carers to co-develop the information sheet. The information sheet included details on the study including the potential risks and benefits of taking part, the ways in which participants could take part in the study (e.g. online via SelectSurvey or through the completion and return of paper versions of the questionnaire), and provided potential participants with the contact details of researchers should they wish to discuss their involvement prior to taking part. All participants responded affirmatively to the question “I have read and understood the participant information sheet” and consent was implied by the completion and return of questionnaires. The study and all associated procedures were approved by the London–West London and Gene Therapy Advisory Committee (GTAC) Research Ethics Committee (16/LO/0386) in February 2016.

### Data analysis

We fitted data from nascent scale to the partial credit “Rasch” model (PCM)[13,14] in order to assess psychometric performance. We evaluated factor structure, scalability and monotonicity by fitting data to non-parametric Mokken model before more rigorous psychometric assessments using the PCM.[15] The combination of the two methodologies has been shown to be useful in previous research conducted by members of our group and others.[16–19] Where scale data did not conform to the assumptions of either the Mokken or the partial credit model, an iterative process of item reduction was undertaken to remove the violating items from the analysis.[20] The iterative process involved assessments of scalability, model and item fit to the PCM, category threshold disordering, local dependency, and differential item functioning (DIF). Each concept and the method by which it is assessed is described in greater detail below.

#### Mokken analysis

The Mokken model is a non-parametric extension of the simple deterministic Guttman scaling model. [21] The model provides a framework to extend the unreastically error-free Guttman models using probabilistic estimation, thus accounting for measurement error.[22] As a non-parametric item response theory (NIRT) model, the Mokken models relax some assumptions of item response theory whilst affirming essential assumptions such as unidimensionality and scalability.[22] We fitted data to the double monotonicity model, a NIRT model which estimates a single parameter for each item (*i*.*e*., the level of the construct which that item assesses). By successfully fitting scale data to a Mokken model it can be said to be both unidimensional and properly scaled. We utilized parallel polychoric principal component analysis which compared the experimental eigenvalues with a Monte Carlo simulated eigenvalues to verify the unidimensional factor structure before proceeding to item response theory analysis.[23,24]

#### The partial credit model

The PCM is a measurement model which describes the probabalistic relationship between the assessment and the respondent as an interaction between the amount of the latent construct that the respondent has (*i*.*e*. involvement with physical health care planning) and the level of the latent construct which the item measures. Both the amount of the construct that the respondent has and the level of the latent construct that the item measures can be described in terms of theta (θ). For example, a item which measures a very high level of physical health care planning (which would be an question that we would not expect many people to affirm; for example the questionnaire item may ask about service user or carer’s access to a document containing a detailed strategy for physical health care) would be less likely to be affirmed than an item measuring a low level of the latent construct (which would be a question that we would expect many people to affirm; for example the questionnaire item may ask about whether a health care professional had asked whether a service user was receiving any care for physical health issues).

Goodness-of-fit statistics can be used to assess the data’s fit to the PCM model at both the item and scale level. In this study we used both the Chi-square and root-mean square error of approximation to evaluate the fit to the model. We accepted both a non-significant Chi-square interaction (P > .05) and RMSEA (< .05) indicating good fit. [25]

#### Category threshold ordering

In the case of a Likert or ‘multiple choice’ item response the probability of responding to each category is modelled separately. As the level of the underlying construct (*i*.*e*., involvement in physical health care planning) rises the probability of responding to each Likert category rises to a peak before falling. Different probabilities are given for each response category at every level of θ. It is essential that each category becomes the most likely response option at a certain level of θ. If this is not the case the item is said to exhibit category threshold disordering.

Category threshold disordering refers to the situation in which one or more of the Likert response categories are not the most likely response at any point of along the underlying θ continuum. In the case of disordered category thresholds, we ‘collapsed’ adjacent categories so that they received the same score. Care was taken not to collapse categories if it were semantically illogical to do so, (*i*.*e*., “Agree” would not be collapsed into “Neither Agree nor Disagree”). An illustrated side-by-side example is provided in a previous paper from our group. [26]

Item response theory models (of which the Rasch model is a special case) are predicated on the assumption that differences in responses to items are driven solely by changes in the underlying trait.[27] One way in which items can violate this assumption is local dependency, a situation whereby the response to one item is dependent on the response to another.[28] In practice, this can occur where items are too similar. Local dependency is assessed using Yen’s Q3 statistic, in which the correlation of item residuals are compared, and item pairs with residual correlations beyond a threshold are said to be locally dependent. We set the threshold to be equal to .2 + the average observed residual.[29,30]

Local dependency can be resolved in a number of ways, including subtesting (where locally dependent items are joined into a ‘super’ item) and item deletion.[31] As we began with a large bank of candidate items, we elected to remove items which were locally dependant. Our strategy was to remove an item if it were locally dependent with more than one other item and then, in the case that a locally independent item pair only demonstrated dependency with one another, item information curves for each item were examined alongside the item wording and the item which provided less information was removed from further analysis.

Another issue which can interfere with the assumption that differences in item scores ought to be driven solely by differences in the underlying trait is differential item functioning (DIF).[32] Differential item functioning occurs when the probability of a certain response to a question varies across different demographic groups. For example, if men were more likely to respond affirmatively to a certain item than women despite having an equal level of overall involvment with physical health care planning, that question would be said to be affected by DIF. We used the iterative hybrid ordinal logistic regression/item response theory approach to conduct DIF analyses. For items flagged as having signficiant DIF following Bonferroni correction, we used the McFadden pseudo R2 estimation with recommend cut-off of R2 > .035 being indicative of meaningful DIF.[33] By assessing DIF between service users and carers we will explore the suitablility of the nascent PREM for both groups. Models were fitted with missing data present. However, missing data were imputed using IRT-based estimation.[34] Given the well-documented issues with model fit statistics, we prioritized meeting the assumptions of the Mokken and Rasch models over model fit, as has been recommended elsewhere.[35]

Items that violated any of the above assumptions were removed, and the remaining items were reanalyzed. We evaluated the reliability of the final scale and the overall fit to the Rasch model. Once a final set of purified questions were calibrated by fitting them to the PCM, we simulated computerized adaptive tests (CATs)[36]. The CAT algorithms conducted stepwise assessments by iteratively selecting the item which will maximise the test information based on the participant based on participant’s θ estimate which is based off their previous responses. The first item for the assessment is the item which maximises information at the mean population level of θ.

We simulated CATs to assess the viability of brief assessment using the nascent scale using the final items as a ‘bank’ of candidate items. In computerized adaptive testing an algorithm is used to select the next most appropriate item for the patients based on their previous responses. This approach has been shown to substantially reduce the length of tick-box assessments whilst maintaining, and even increasing, reliability.[18,26]

We similated CATs using the Firestar script for R. Firestar uses a Bayesian expected a posteriori θ estimator and selected items based on the maximum posterior weighted information (MPWI) criterion. The MPWI selects items based on the item information weighted by the posterior distribution of trait/phenomena values.[37]This criterion has been shown to provide excellent measurement information for CAT using polytomous items.

### Software

Analyses were conducted using the R Statistical Computing Environment with the ‘mokken’, ‘mirt’, ‘lordif’, ‘psych’, ‘ggplot2’, ‘methcomp’ and ‘BlandAltLeh’ packages installed. [34,38–42]. Computer adaptive testing simulation was conducted using the FIRESTAR script, which was modified to add additional statistics.[43] This modified FIRESTAR code is available on request from the authors.

## Results

We collected data from 267 mental health services users from the United Kingdom. 67 participants completed the 67 candidate questionnaire items a second time after two weeks. No data were available on the number of participants who began the survey but did not complete it. 16% of PREM data was missing. Demographic information is displayed in Table 1.

**Table 1:** Demographic information for 267 participants recruited into the study.

Age	44(14)	
Gender	69.3% Female	
	21% Male	
	9.7% unreported	
Ethnicity		
	83.7% White	
	16.3% Non-white	
Service user/carer status		
SU	66%	
Carer		15%
Both SU and carer		12%
Not reported		7%
Geographic location		
Northern England		32%
The Midlands		23.7%
Southern England		39.4%
Ireland		1%
Scotland		1.5%
Wales		2.5%

Key: SU = Service users

### Mokken analysis

Mokken analysis revealed violations of monotonicity for a number of items (5, 6, 8, 10, 25, 26, 40). In addition, Loevinger’s scalability coefficient was too low (Item H >.30) for items 7, 9, 39. The 57 remaining items were free from violations of monotonicity and were unidimensional. Parallel principal component and factor analysis confirmed the unidimensional structure of the dataset as the eigenvalue for the second factor/component (2.87, 2.16) was below simulated eigenvalues in the Monte Carlo dataset (1.50, 1.19).

### Rasch analysis

The remaining items were fitted to the partial credit model. The initial fit to the model was poor (RMSEA > .10); thus prompting evaluation of item performance in the context of Rasch model assumptions. There appeared to be substantial threshold disordering throughout the scale. A single solution was chosen to rescore all items (0-1-1-2-2). The amended threshold probability curves for all the items can be see in S1 Table.

**Fig 1:**
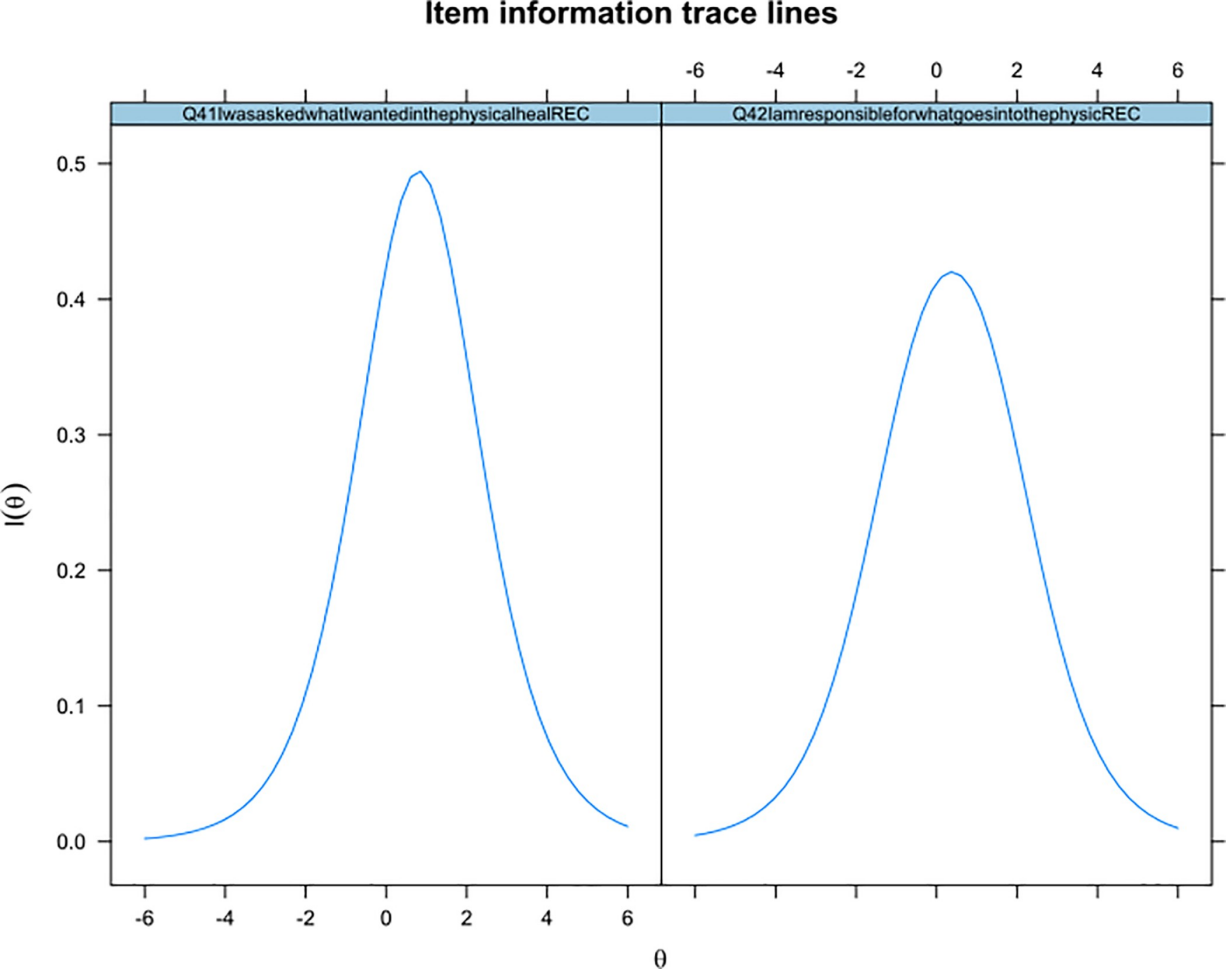
Comparison of item information curves for locally dependent items.

Model fit improved slightly after rescoring but was still unacceptable (RMSEA = .097 (95% CI = .091-.10)). We evaluated the correlations between item residuals, which were above the threshold in a number of instances. In total, 96 item pairs were locally dependant (see S2 Table). A total of 27 individual items that displayed local dependency with more than one other item were removed from the analysis. Four sets of items remained which were locally dependant with one another. The item information curves for both pairs of locally-dependent items were compared side-by-side (see Fig 1) and in each case the item with the lowest item information removed from the scale.

Fig 1 Shows item information curves for a pair of locally-dependent items. The amount of information which each gives about the participant is given on the *y*-axis and the level of underlying construct that the person has (i.e., involvement with physical health care planning) is on the *x-*axis.

No DIF was detected for age or gender but item 65 “My care planning team communicates effectively” was found to have significant DIF (R2 change = .06) between service users and carers.

Following adjustment for category threshold ordering, local dependency, and differential item functioning; item 36 “I feel comfortable attending discussions about my care plan” misfit the model and was removed (x^2^ = 34.81, df = 15, *P* = .003). The removal of item 36 led to a final item bank of 19 items which were free from breaches of assumptions of the Rasch model, displayed excellent model fit (x^2^ = 192.94, df = 1515, *P* = .02, RMSEA = .03 (95% CI = .01-.04). The 19-item bank had excellent reliability (marginal r = 0.87). Details of final items, including parameters, are given in Table 2.

**Table 2:** Details of final items and item threshold parameters.

	Final items										
Item Number	Original number	Wording	Model fit			Item Threshold Parameters		item fit statistics			
			*χ*^2^	df	P	delta 1	delta 2	*χ*^2^	df	P	Scoring
1	50	My care planning team ask about my existing physical health conditions.	21.81	#	.11	-1.20	.12	31	#	0.6	0-1-1-2-2
2	24	The physical health information in my care plan is personalised.	13.29	#	.72	-1.92	.20	38	#	0.3	0-1-1-2-2
3	53	My care planning team encourage me to take responsibility for my physical health care planning.	14.10	#	.59	-2.01	.27	25	#	0.8	0-1-1-2-2
4	37	My opinion on my physical health is valued by my care planning team.	13.48	#	.64	-2.03	.38	27	#	0.7	0-1-1-2-2
5	4	I know who reads the physical health information contained within my care plan	11.79	#	.69	-1.02	.43	27	#	0.7	0-1-1-2-2
6	55	My care planning team offer practical advice about my physical health.	15.44	#	.42	-1.42	.51	33	#	0.4	0-1-1-2-2
7	13	My care plan gives details of my physical health history.	20.20	#	.16	-1.16	.52	27	#	0.7	0-1-1-2-2
8	15	My thoughts about my physical health are included in my care plan.	11.20	#	.85	-.62	.76	28	#	0.5	0-1-1-2-2
9	52	I experience continuity of care for the treatment of both my physical health conditions and mental health conditions.	15.22	#	.58	-.68	.77	40	#	0.2	0-1-1-2-2
10	22	The physical health information in my care plan is helpful.	9.43	#	.97	-1.61	.83	28	#	0.7	0-1-1-2-2
11	16	Physical health reviews are carried out in a timely manner.	14.55	#	.56	-.53	.86	28	#	0.7	0-1-1-2-2
12	62	My care planning team have a good understanding of my fears about future physical health conditions.	15.30	#	.64	-.77	.92	28	#	0.6	0-1-1-2-2
13	56	My care planning team have the time they need to talk to me about physical health concerns.	28.39	#	.08	-1.18	.94	27	#	0.5	0-1-1-2-2
14	44	The content of my physical health care plan is responsive to changes in my circumstances.	15.06	#	.72	-1.14	1.06	26	#	0.8	0-1-1-2-2
15	27	Information in my care plan has helped me to maintain my physical health.	13.12	#	.78	-.63	1.08	26	#	0.8	0-1-1-2-2
16	41	I was asked what I wanted in the physical health information in my care plan.	14.07	#	.59	-.25	1.13	32	#	0.5	0-1-1-2-2
17	60	The care plan adequately addresses any side effects I experience from my medication.	14.79	#	.74	-1.22	1.13	40	#	0.3	0-1-1-2-2
18	46	I have had the opportunity to invite all the relevant people to care planning meetings related to my physical health.	17.23	#	.37	-.55	1.27	32	#	0.5	0-1-1-2-2
19	51	My care planning team makes sure my mental health is not prioritised over my physical health.	21.01	#	.34	-1.15	1.51	32	#	0.5	0-1-1-2-2

### Test-retest reliability

**Fig 2:**
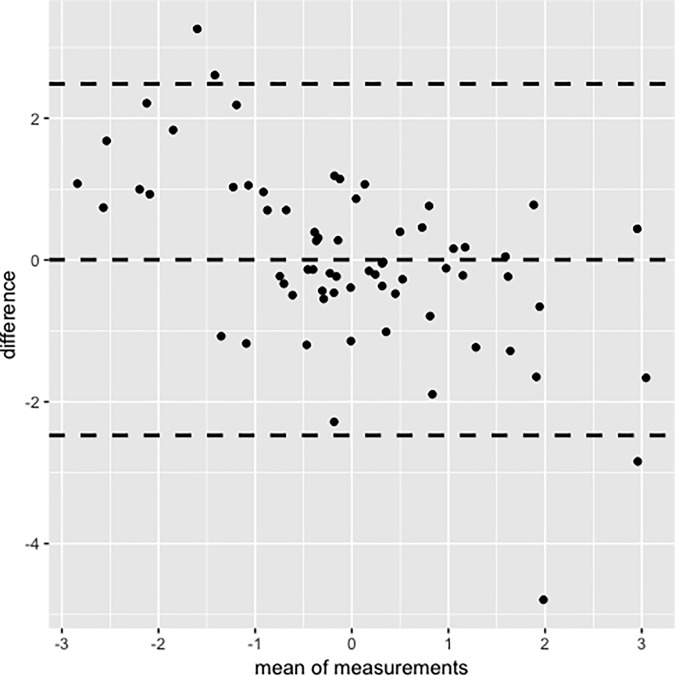
Bland Altman plot for test-retest reliability.

The correlation between theta scores at baseline and 2-week follow-up was high (r = .70, *P* < .01). Bland Altman analysis revealed that 94.9% of assessment pairs were within the 95% limits of agreement (see Fig 2).

### Computerized adaptive testing

**Fig 3:**
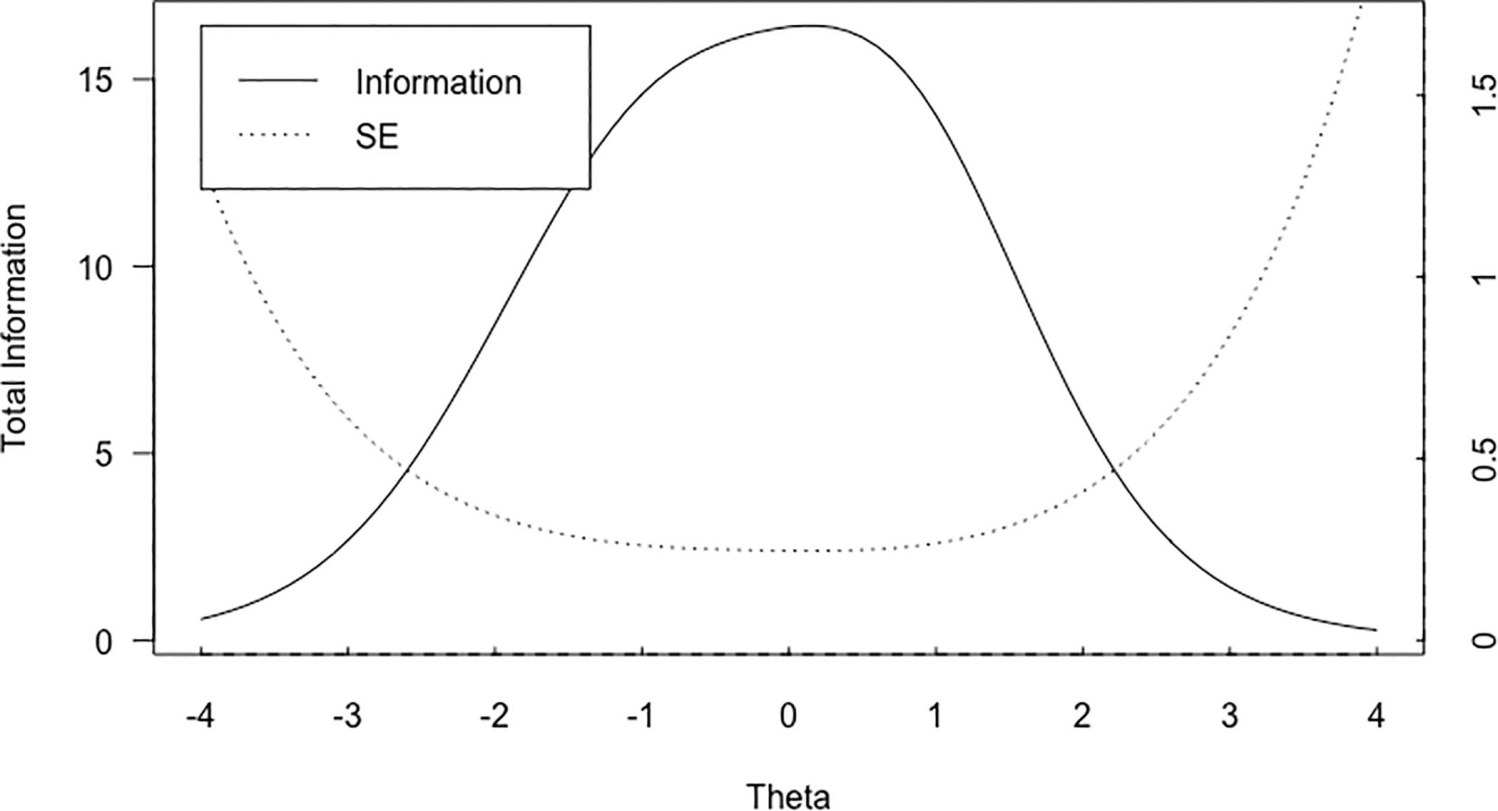
Overall scale information and standard error. Key–SE = Standard Error.

Adaptive testing simulations were conducted with a simulated Gaussian N (-0.08,1.90) distribution, which matched the distribution of the data used to develop the item banks. Results of CAT simulation are shown in the Table 3. Assessments as short as 10 items demonstrated high correlation with the total score of the full scale. The overall information and standard error which was available in the entire item banks is displayed in Fig 3.

**Table 3:** Summary of simulated computer adaptive tests.

	Standard Error (SE)			
Number of items	Mean	SD	Range	Correlation with full scale
19	.36	.04	.32-.56	1
15	.41	.04	.38-.60	.98
10	.43	.04	.40-.65	.95
5	.66	.04	.62-.78	.87

## Discussion

We present the co-development and validation of a new service user and carer-reported assessment for physical health care planning in serious mental health services, the EQUIP Physical Health PREM (EQUIP-PH-PREM).

The new PREM contains 19 items which were successfully fitted to a single-parameter Rasch item response theory model. The PREM is suitable for assessing both service users and carers. The 19 items also serve as an item bank for computerized adaptive testing (CAT) which can tailor assessments to the individuals who complete the PREM. We show that using CAT administration could substantially reduce burden of response by reducing the number of items in the assessment from 19 to 10, whilst still maintaining acceptable accuracy and high correlation between scores.

In the EQUIP-PH-PREM we provide a tool to support investigations into the experience of service users and carers who are receiving care for a severe mental illness from mental health providers. Adequate service user and carer involvement in care planning decisions are predicated on successful interaction both within and between stakeholder groups. In order to ensure the new PREM incorporated these important aspects the items were developed in collaboration with service users, carers and mental health professionals.

The final PREM items include those that cover having the opportunity and time to be able to discuss physical health concerns, reflecting previously identified organisational barriers to providing integrated care.[12] Similarly, they highlight the importance of *co-created* care plans, which are known to be highly valued by both service users and carers [44,45] Further items serve to facilitate long-term self-management skills that are required to manage physical health concerns.

This new PREM has operationalized the evidence-based best practice framework developed previously which will allow health care providers and service users to challenge current practice by quantifying service user and carer involvement from the user perspective.[12]

The measure will facilitate benchmarking of service quality and service user experience, aligned with contemporary philosophies and policies for collaborative recovery-focused mental health care. The philosophy of the new PREM is that mental and physical health are equally important (the so-called parity of esteem), and parity of esteem is increasingly being embedded in policy and practice imperatives derived from stakeholder consultation.[46]

The EQUIP-PH PREM assesses issues which have been consistently highlighted in consultations with service users and carers and, as such, is well suited for use as an tool to assess the outcome of interventions. Other relevant interventions include those designed to improve inter- and intra- professional communication including professional training and improved health systems to enhance the integration and continuity of care for those under the care of health services.

The current study has some limitations. Firstly, our dataset consisted of predominantly white, female service users. Though all systematic differences between demographic groups were corrected for in the current analysis, further research would be warranted to ensure that the items perform well in groups which were not well represented in our data. It should be noted that whilst we demonstrated uniform scale performance across demographic groups–including service users and carers, we did not collect information relating to comorbidities, physical activity or substance and further research would be necessary to explicitly confirm that the scale is unaffected by differences in disease or lifestyle factors within groups of service users.

Our study is also limited by the necessity to evaluate the CATs using simulated, rather than actual, data. This technique is likely to the slightly over-estimate the accuracy of the CAT as it does not take into account aberrant responders who do not conform to the expectations of the model. Our previous research developing item banks for depression and quality of life suggests that this effect is marginal and that CAT assessment is efficient and precise both when simulations are made using participant data and when the CAT is deployed in the real world.[18]

It is noteworthy that when administering CATs each individual respondent is likely to complete different combinations of items which form a subset of the complete item bank. Though the scores between the unidimensional CAT and the fixed-length short-form are highly correlated, there is no guarantee that every patient will complete items from each of the content domains which were nominated by service users and carers. In the current manuscript, we prioritize brevity and accuracy and simulate CAT administration without content balancing or prioritizing certain items. We acknowledge that other users may prioritize item exposure and thus may utilize CATs differently.

Parties who wish to use CAT administration for the EQUIP-PH measure are directed towards many packages available for the R Statistical Programming Environment including mirt and catR.[34,47] One tool for implementing CATs is the Concerto platform, developed and maintained by the University of Cambridge.[48] Further details can be found on the Concerto website (concertoplatform.com) or by request to the authors of this manuscript.

Our study also has some notable strengths. We have collected a geographically diverse group of both service users and carers and created a flexible assessment which can be used without modification of assessing and comparing both groups. The EQUIP-PH PREM which we have developed is related to the EQUIP measure, a questionnaire measure for service user and carer involvement in care planning, which was recently developed by our group[9]. Both tools could be used together to gain a holistic understanding of how involved service users and carers are in mental health care planning. Further research could usefully be conducted to understand the scores from the two instruments in relation to one another and provide further insight into their use as a tool to assess global care planning and service delivery.

In conclusion, The EQUIP-PH PREM is a brief, accurate, and flexible service user- and carer-reported assessment of involvement in physical health care planning for users of mental health services with serious mental illnesses. The measure provides a reliable means to evaluate and benchmark the quality of physical health management in the context of mental health care.

## Supporting information

S1 Table Anonymized baseline dataset. (CSV)

S2 Table Anonymized follow up dataset. (CSV)
